# Estratégia Farmacoinvasiva em Idosos até 75 Anos ou Não Idosos: Análise de Parâmetros Bioquímicos e de Ressonância Nuclear Magnética Cardíaca

**DOI:** 10.36660/abc.20220177

**Published:** 2022-12-20

**Authors:** Amanda S. Bacchin, Francisco A. H. Fonseca, Rui Povoa, Gilberto Szarf, Ibraim Masciarelli Pinto, Adriano Mendes Caixeta, Daniela Teixeira, Ieda Longo Maugeri, Mayari E. Ishimura, Maria E. R. Coste, Henrique Tria Bianco, Carolina N. França, Maria Cristina Izar

**Affiliations:** 1 Universidade Federal de São Paulo São Paulo SP Brasil Universidade Federal de São Paulo, São Paulo, SP – Brasil; 2 Escola Paulista de Medicina Universidade Federal de São Paulo Santana de Parnaíba SP Brasil Escola Paulista de Medicina da Universidade Federal de São Paulo, Santana de Parnaíba, SP – Brasil; 3 Instituto Dante Pazzanese de Cardiologia São Paulo SP Brasil Instituto Dante Pazzanese de Cardiologia – Cardiovascular CT/MR, São Paulo, SP – Brasil; 4 Universidade de Santo Amaro São Paulo SP Brasil Universidade de Santo Amaro – Pós-Graduação, São Paulo, SP – Brasil

**Keywords:** Infarto do Miocárdio com Supradesnível do Segmento ST, Citocinas, Linfócitos

## Abstract

**Fundamento:**

A estratégia farmacoinvasiva é uma alternativa na inviabilidade da intervenção coronária percutânea primária (ICP).

**Objetivos:**

Este estudo teve como objetivo avaliar os efeitos da estratégia farmacoinvasiva precoce sobre o tamanho da área infartada e a fração de ejeção ventricular esquerda em pacientes idosos e não idosos. O papel dos marcadores inflamatórios também foi avaliado.

**Métodos:**

Pacientes (n=223) com infarto do miocárdio com elevação do segmento ST (IAMCSST) foram prospectivamente incluídos e submetidos à trombólise medicamentosa nas primeiras seis horas, e à angiografia coronariana e à ICP, quando necessária, nas primeiras 24 horas. As amostras de sangue foram coletadas no primeiro dia (D1) e 30 dias após (D30). A ressonância magnética cardíaca foi realizada no D30. O nível de significância estatística foi estabelecido em p<0,05.

**Resultados:**

Pacientes idosos e não idosos apresentaram porcentagem similares de massa infartada [13,7 (6,9-17,0) vs. 14,0 (7,3-26,0), respectivamente p=0,13)] [mediana (intervalo interquartil)]. No entanto, os pacientes idosos apresentaram maior fração de ejeção ventricular esquerda [53 (45-62) vs. 49 (39-58), p=0,025)]. As concentrações de interleucina (IL)1beta, IL-4, IL-6, e IL-10 não foram diferentes entre D1 e D30, mas pacientes idosos apresentaram níveis mais elevados de IL-18 em D1 e D30. O número absoluto de linfócitos B e T foram similares em ambos os grupos em D1 e D30, porém, pacientes idosos apresentaram uma razão neutrófilo-linfócito mais alta em D30. A análise de regressão linear multivariada dos desfechos de RMC de toda a população do estudo mostrou que os preditores independentes não foram diferentes entre pacientes idosos e não idosos.

**Conclusão:**

A estratégia farmacoinvasiva em pacientes idosos foi associada a pequenas diferenças nos parâmetros inflamatórios, tamanho do infarto similar, e melhor função ventricular esquerda em comparação a pacientes não idosos

## Introdução

A mortalidade por infarto do miocárdio com elevação do segmento ST (IAMCSST) é mais ata em pacientes idosos que em pacientes não idosos.^1 , 2^ Atrasos no diagnóstico e na terapia trombolítica ou percutânea resultam em maior perda do miocárdio e remodelamento ventricular, com implicações no prognóstico.^3 , 4^ A maior prevalência de fatores de risco cardiovascular, tais como diabetes e hipertensão, e doença coronariana grave aumenta o desafio no tratamento de pacientes idosos.^5 - 7^

Estratégias farmacoinvasivas surgiram como uma grande oportunidade de reduzir o tempo para reperfusão coronária, permitindo uma janela adequada para tratamento cirúrgico ou percutâneo complementar.^8 , 9^ Ainda, um suprimento sanguíneo mais eficiente promovido por vasos colaterais pode aumentar a recuperação do miocárdio isquêmico.^10^ No entanto, devido à imunossenescência, respostas inflamatórias mais intensas em pacientes idosos podem exercer um papel adicional durante a reperfusão e recuperação do miocárdio.^11 , 12^ Finalmente, em longo prazo, após o IAMCSST, a recorrência de eventos coronarianos e mortes por doenças cardiovasculares parecem estar relacionadas à extensão da necrose no miocárdio e o grau de disfunção ventricular.^13 , 14^

Nosso estudo teve como objetivo comparar a eficácia da estratégia farmacoinvasiva na quantidade de massa infartada e na função ventricular, avaliadas por ressonância magnética cardíaca (RMC) em idosos (até 75 anos de idade) e não idosos com IAMCSST. O estudo também avaliou o papel de marcadores inflamatórios nesses parâmetros de RMC.

## Métodos

### População do estudo

Foram incluídos pacientes consecutivos de ambos os sexos em seu primeiro episódio de infarto do miocárdio. Todos os pacientes foram submetidos à trombólise medicamentosa com tenecteplase nas primeiras seis horas do início dos sintomas, seguido de angiografia coronariana e intervenção coronária percutânea (ICP) quando necessária, nas primeiras 24 horas do IAMCSST. Esses pacientes receberam tratamentos padrões (incluindo agentes hipolipemiantes de alta eficácia e terapia antiplaquetária dupla). Pacientes com instabilidade clínica, doença autoimune, doença maligna, sinais de infecção ativa, ou idade superior a 75 anos (por questões de segurança relacionada à terapia medicamentosa) foram excluídos. O protocolo do estudo seguiu a Declaração de Helsinki e foi aprovado pelo comitê de ética local (CAAE: 38692514.1.1001.5505; IRB 1.253.088). Os participantes assinaram um termo de consentimento antes de serem incluídos no estudo.

### Exames laboratoriais

Todos os pacientes foram encaminhados ao nosso hospital após trombólise para angiografia coronária durante as primeiras 24 horas do infarto do miocárdio. As amostras de sangue foram coletadas no primeiro dia de internação. Nos casos de pacientes transferidos durante a noite, as amostras eram colhidas na manhã seguinte. As amostras foram colhidas no primeiro dia (D1) e após 30 dias de IAMCSST (D30).

Os exames laboratoriais de rotina foram realizados no laboratório central do hospital universitário. As concentrações de proteína C reativa ultrassensível (PCRus) foram medidas por imunonefelometria, e subtipos B e T de linfócitos foram determinados como descrito anteriormente.^12^ Em resumo, as células foram descongeladas e diluídas em meio RPMI. As células foram coradas com anticorpos monoclonais conjugados (com agente fluorescente) para a avaliação de linfócitos B e T. A população de linfócitos B1 foi definida como CD3^-^ CD19^+^ CD20^+^ CD27^+^ CD43^+^, e a de linfócitos B2 como CD3^-^ CD19^+^ CD20^+^ CD43^-^. Os linfócitos T foram definidos como CD4^+^ (CD3^+^ CD4^+^ CD8^-^) ou CD8^+^ (CD3^+^ CD4^-^ CD8^+^).

Os níveis de interleucinas circulantes foram determinados por ensaio de imunoabsorção enzimática (ELISA) utilizando kits específicos para IL-4, IL-6 e IL-10 (BD Pharmingen-EUA) e R&D Systems kits (Minneapolis, EUA) para IL-1β e IL-18.^15^

### Ressonância magnética cardíaca

A RMC foi realizada no trigésimo dia após o IAMCSST para quantificar a massa infartada e investigar os efeitos das variáveis inflamatórias durante a recuperação do infarto do miocárdio.^16^ As imagens foram realizadas em scanners de 3.0T e o protocolo incluiu a geração de imagens de precessão livre em estado estacionário para a análise anatômica, cine-ressonância retrospectiva em cortes de eixo longo e eixo curto e realce tardio com gadolínio para avaliação de cicatriz/fibrose miocárdica como descrito anteriormente.^12^ A massa de tecido necrótico foi estimada em gramas e em porcentagem de tecido necrótico da massa ventricular esquerda.

### Análise estatística

As variáveis categóricas foram descritas em número e porcentagem e comparadas pelo teste do qui-quadrado de Pearson. As variáveis contínuas foram avaliadas quanto à normalidade pelo teste de Kolmogorov-Smirnov e expressas em mediana [intervalo interquartil (IIQ)] devido à sua distribuição não normal. O teste não paramétrico de soma dos postos de Wilcoxon foi usado para comparar as variáveis contínuas em D1 e em D30, e o teste de Mann-Whitney para comparar as variáveis contínuas entre pacientes idosos e não idosos. As análises de regressão linear univariada e multivariada foram realizadas para identificar preditores independentes para a massa infartada e fração de ejeção ventricular esquerda (FEVE). Foram analisadas todas as premissas para a análise de regressão linear, e uma amostra por conveniência foi utilizada. A análise estatística foi realizada pelo programa SPSS versão 17.0 e um valor de p<0,05 foi definido como estatisticamente significativo.

## Resultados

**Figura 1:**
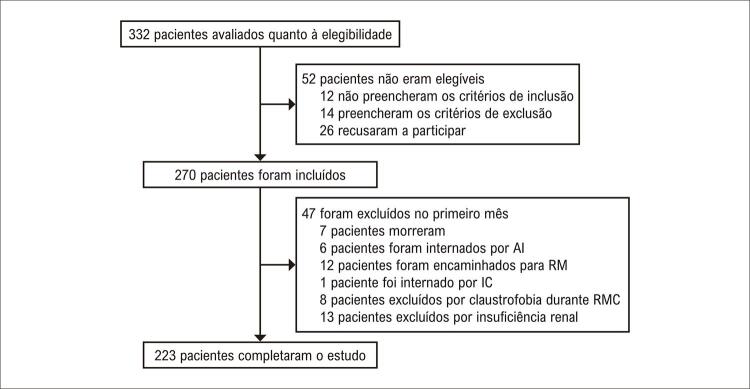
Fluxograma do estudo. Foram incluídos pacientes estáveis, após primeiro infarto do miocárdio. Cinquenta e dois pacientes foram elegíveis e 47 excluídos por morte, internações ou contraindicação para ressonância magnética cardíaca. Um total de 223 idosos e não idosos completaram o estudo e se submeteram à RMC após 30 dias do infarto agudo do miocárdio. AI: angina instável; RM: revascularização miocárdica cirúrgica; IC: insuficiência cardíaca; RMC: ressonância magnética cardíaca.

Um total de 332 pacientes com IAMCSST foram rastreados, e 270 incluídos no estudo entre maio de 2015 e março de 2019. Quarenta e sete pacientes foram excluídos das análises de RMC no primeiro mês devido à mortalidade, internação ou insuficiência renal ( Figura 1 ). No primeiro mês, a taxa de eventos cardiovasculares compostos por reinternações por insuficiência cardíaca, angina instável, morta súbita ressuscitada, ICP de urgência, e mortalidade total não foram diferentes entre os grupos (p=0,49, teste do qui-quadrado). Todos esses eventos cardiovasculares apresentaram distribuição similar entre idosos e não idosos, com exceção para mortalidade quatro pacientes idosos e três pacientes no grupo de não idosos (p=0,017, teste do qui-quadrado). Outros eventos cardiovasculares tais como ICP eletiva, revascularização cirúrgica eletiva e sangramento não foram diferentes entre esses grupos.

A Tabela 1 apresenta as principais características da população estudada (n=223). O local do infarto do miocárdio, níveis de troponina T ultrassensível (TNTus), fluxo coronariano (fluxo TIMI), vasos colaterais (classificação Rentrop) e microcirculação (grau de blush miocárdico) foram similares em ambos os grupos.

**Tabela 1:** Características principais da população do estudo

Parâmetros	Não idosos (<65 anos) N=180	Idosos (65-75 anos) N=43	Valor p
Idade, anos	54 (48-59)	67 (66-70)	<0,001
Sexo masculino	131 (73)	27 (63)	0,18
Diabetes	47 (26)	10 (23)	0,82
Hipertensão	92 (51)	17 (40)	0,85
Tabagismo	86 (48)	14 (33)	0,004
Índice de massa corporal, kg/m^2^	26,6 (25,6-29,7)	25,5 (23,1-28,6)	0,13
PAS, mm Hg	124 (110-137)	130 (121-140)	0,10
PAD, mm Hg	77 (70-90)	78 (70-86)	0,98
TFG, mL/min/1,73m^2^	93,5 (81,0-100,0)	77,0 (65,0-88,0)	<0,001
TNTus, U/L	5037 (2238-10089)	4298 (1161-11673)	0,36
**Tipo de IM**			**0,41**
Anterior	88 (52)	17 (40)	
Inferior	70 (41)	22 (51)	
Lateral	11 (7)	4 (9)	

*Valores em mediana (intervalo interquartil IQR) ou frequências (%); PAS: pressão arterial sistólica; PAD: pressão arterial diastólica; TFG: taxa de filtração glomerular (estimada por CKD-EPI); TNTus: troponina T ultrassensível; IM: infarto do miocárdio. As variáveis contínuas foram comparadas pelo teste de Mann-Whitney; variáveis categóricas foram comparadas pelo teste de qui-quadrado de Pearson.*

### Parâmetros laboratoriais

A Tabela 2 mostra que indivíduos idosos e não idosos apresentaram parâmetros laboratoriais comparáveis, exceto pelos níveis mais altos de HDL colesterol, razão neutrófilo-linfócito (RNL) e creatinina sérica no grupo de idosos.

**Tabela 2:** Parâmetros laboratoriais no basal (D1) e após 30 dias (D30), por grupo

Parâmetros	Não idosos (< 65 anos) N=180	Idosos (65-75 anos) N=43	Valor p
**D1**			
Leucócitos (células/mm^3^)	12000 (9640-13675)	11000 (9160-12300)	0,07
Linfócitos	2098 (1532-2725)	1647 (1298-2558)	0,11
RNL	4,63 (3,10-6.78)	4,74 (3,20-7,47)	0,80
Glicose	121 (99-151)	119 (107-142)	0,75
HbA1c	5,9 (5,6-6,6)	6,0 (5,5-6,5)	0,73
Colesterol total	198 (173-230)	198 (166-232)	0,75
Colesterol LDL	129 (107-154)	123 (103-154)	0,57
Colesterol HDL	41 (33-46)	44 (39-55)	0,03
Triglicerídeos	133 (97-203)	113 (79-172)	0,10
Colesterol não HDL-C	159 (136-193)	151 (120-175)	0,30
Lp (a)	16 (9-41)	19 (13-46)	0,20
Creatinina	0,86 (0,74-1,01)	0,91 (0,78-1,10)	0,06
TFG	94 (81-100)	77 (65-88)	<0,001
PCRus	21,9 (9,5-48,1)	18,1 (10,8-33,2)	0,63
**D30**			
Leucócitos	7740 (6500-9050)	7710 (6630-8830)	0,94
Linfócitos	2044 (1590-2441)	1823 (1468-2210)	0,06
RNL	2,77 (2,25-3,48)	3,50 (2,52-4,23)	0,04
Glicose	99 (90-112)	99 (92-115)	0,91
Colesterol total	123 (106-146)	121 (105-156)	0,85
Colesterol LDL	61 (46-83)	61 (47-84)	0,93
Colesterol HDL	37 (31-44)	39 (34-47)	0,23
Triglicerídeos	127 (97-164)	128 (104-163)	0,97
Colesterol não HDL-C	84 (67-109)	84 (69-113)	0,74
Lp (a)	14 (8-37)	16 (11-38)	0,20
Creatinina	0,93 (0,82-1,08)	1,05 (0,86-1,22)	0,02
TFG	85 (73-87)	68 (59-79)	<0,001
PCRus	1,99 (0,88-5,27)	3,03 (1,35-7,29)	0,08

*Valores em mediana (IIQ); RNL: razão neutrófilo-linfócito; HbA1c: hemoglobina glicada (%); PCRus: Proteína C Reativa ultrassensível (mg/L); TFG: taxa de filtração glomerular (CKD-EPI, mL/min/1,73m^*2*^). Lp (a): lipoproteína (a). Lipídios, glicose e creatinina expressos em mg/dL; Comparações pelo teste de Mann-Whitney.*

No D1 e no D30, os números de linfócitos T CD4, CD8, bem como linfócitos B (subtipos B1 ou B2), foram comparáveis nos grupos de idosos e não idosos. Além disso, o número absoluto de linfócitos B2 clássicos (naïve e de memória) não foi diferente entre os grupos ( Tabela 3 ).

**Tabela 3:** Linfócitos B e T no basal (D1) e após 30 dias (D30)

Parâmetros	Não idosos (< 65 anos) N=180	Idosos (65-75 anos) N=43	Valor p
**D1**			
T CD4	871 (567-1198)	1076 (597-1625)	0,40
T CD8	345 (226-484)	307 (188-799)	0,99
B1	5,0 (3,0-11,0)	5,5 (3,3-13,0)	0,58
B2 naïve	53,4 (16,0-115,9)	52,2 (9,89-115,4)	0,58
B2 de memória	49,9 (23,4-129,0)	59,5 (27,1-113,3)	0,72
B2 clássico	135,1 (75,6-248,3)	124,6 (81,5-226,8)	0,84
**D30**			
T CD4	912 (620-1093)	976 (730-1204)	0,23
T CD8	313 (197-479)	310 (197-552)	0,80
B1	4,2 (2,4-8,1)	2,6 (2,0-5,0)	0,23
B2 naïve	38,5 (11,6-75,0)	59,9 (15,5-104,5)	0,22
B2 de memória	38,6 (20,1-85,6)	57,7 (35,9-94,6)	0,07
B2 clássico	97,4 (53,6-159,6)	122,0 (87,4-195,6)	0,08

*Linfócitos em células/mL; B2 clássico = B2 naïve + B2 de memória; comparações pelo teste de Mann-Whitney.*

**Figura 2:**
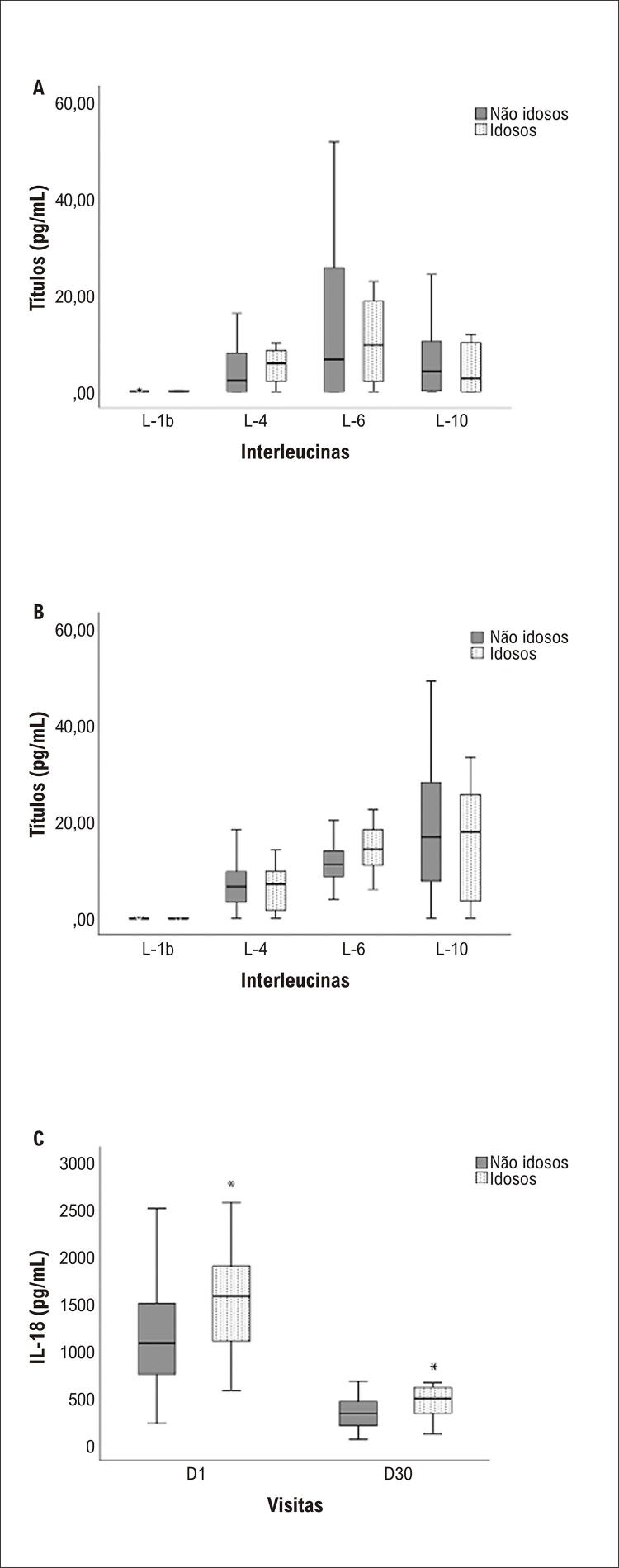
Boxplots de interleucinas circulantes (IL) no basal (D1) e 30 dias após o infarto agudo do miocárdio (D30). (A) Concentrações similares de IL-1β, IL-4, IL-6 e IL-10 foram observadas para pacientes idosos e não idosos em D1; (B) Em D30, concentrações comparáveis de IL-1β, IL-4, IL-6 e IL-10 foram observadas; (C) As concentrações de IL-18 eram mais elevadas nos idosos em D1 (p=0,017 vs. não idosos), e D30 (p=0,023 vs. não idosos). Todas as análises foram realizadas pelo teste de Mann-Whitney U *diferenças significativas.

Em comparação ao D1, foram observados níveis mais elevados de IL-4 (p=0,007) e IL-10 (p<0,001), níveis mais baixos de IL-18, e nenhuma mudança significativa para IL-1 beta (p=0,058) ou IL-6 (p=0,77). Diferenças entre idosos e não idosos em D1 e D30 foram detectadas somente para IL-18 ( Figura 2 ).

### Ressonância magnética cardíaca (RMC)

**Figura 3:**
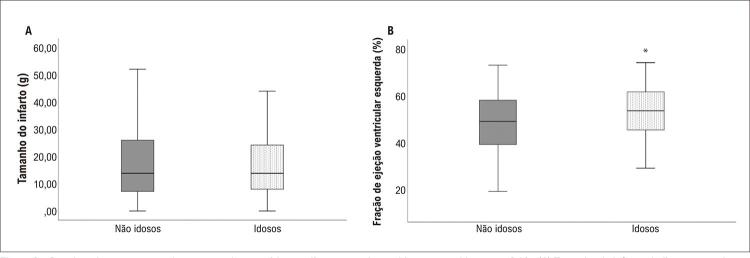
Boxplots dos parâmetros de ressonância magnética cardíaca em pacientes idosos e não idosos em D30. (A) Tamanho de infarto similar entre pacientes idosos e não idosos (p=0,25); (B) Maior fração de ejeção do ventrículo esquerdo (FEVE) nos idosos (p=0,02); teste de Mann-Whitney; *diferenças significativas.

A Figura 3 apresenta os parâmetros de RMC de pacientes idosos e não idosos. O tamanho do infarto foi comparável entre os grupos, e uma FEVE maior foi observada nos idosos.

A massa do ventrículo esquerdo (VE) (gramas) [105 (82-122) vs. 103 (85-123) p=0,78) para idosos e não idosos respectivamente], bem como a porcentagem de fibrose do VE [13,7 (6,9-17,0) vs. 14,0 (7,3-26,0), p=0,13 para idosos e não idosos respectivamente] não foram diferentes entre os grupos.

Tanto a FEVE (%) como a fração de ejeção do ventrículo direito [59 (52,0-67,0) vs. 55,0 (50,0-62,0), p=0,012] no D30 foram mais altas em idosos em comparação a não idosos. A porcentagem de pacientes com FEVE ≤ 40% foi similar em ambos os grupos de pacientes (qui-quadrado 1,39, p=0,24).

### Regressão linear multivariada

A análise univariada revelou que os níveis de TNTus, PCRus, pressão arterial sistólica, e peso foram significativamente associados com o tamanho do infarto (gramas) em D1 ( Tabela 4 ). A análise de regressão linear multivariada (ANOVA p<0,001) identificou TNTus (coeficiente beta -0,367, p<0,001), PCRus (coeficiente beta -0,273, p<0,001), e glicemia (coeficiente beta -0,162, p=0,018) como preditores independentes. Para FEVE, a análise de regressão univariada em D1 mostrou uma associação significativa com níveis de TNTus, PCRus, RNL, leucócitos e linfócitos, e glicemia ( Tabela 4 ).

**Tabela 4:** Análise de regressão linear univariada

	Massa infartada (gramas)		FEVE (%)	

	r	Valor p	r	Valor p
**D1**				
TNTus	0,380	<0,001	0,428	<0,001
PCRus	0,331	<0,001	0,319	<0,001
RLN	0,153	0,038	0,296	<0,001
PAS	0,171	0,022	0,135	0,062
Creatinina	0,152	0,029	0,038	0,575
Peso	0,139	0,046	0,035	0,608
Leucócitos	0,099	0,178	0,231	0,001
Glicemia	0,049	0,586	0,162	0,016
Linfócitos	0,144	0,051	0,178	0,012
**D30**				
Creatinina	0,272	<0,001	0,214	0,002
Leucócitos	0,179	0,015	0,066	0,353
B2 naïve	0,207	0,024	0,154	0,080
T CD4	0,267	0,023	0,134	0,239
T CD8	0,273	0,020	0,265	0,018
Colesterol total	0,101	0,153	0,160	0,019
Colesterol não-HDL	0,048	0,503	0,150	0,032
HDL-C	0,209	0,004	0,160	0,019

*TNTus: troponina T ultrassensível; PCRus: proteína C reativa ultrassensível; linfócitos B e T em células/mL r – coeficiente de correlação; D1: amostras colhidas no primeiro dia do infarto do miocárdio; D30: amostras colhidas 30 dias após infarto do miocárdio; FEVE: fração de ejeção do ventrículo esquerdo. Massa infartada e fração de ejeção ventricular esquerda determinadas por ressonância magnética cardíaca no D30.*

Em D30, a análise de regressão univariada apresentou as seguintes variáveis associadas com o tamanho do infarto: creatinina, linfócitos B2 naïve, linfócitos T CD4, linfócitos T CD8, HDL colesterol, e leucócitos ( Tabela 4 ). A análise de regressão multivariada em D30 (ANOVA p<0,001) identificou a creatinina (coeficiente beta 0,247, p =0,001) e HDL colesterol (coeficiente beta -0,172, p=0,017) como preditores independentes da massa infartada. Para a FEVE, entre as variáveis coletadas em D30, a análise de regressão univariada mostrou associação entre linfócitos T CD8, creatinina, colesterol não HDL, colesterol total ( Tabela 4 ). Após a análise de regressão multivariada, a creatinina permaneceu como preditor independente de FEVE (ANOVA p=0,002, coeficiente beta -0,211).

## Discussão

A estratégia farmacoinvasiva tem sido considerada uma alternativa eficaz à ICP em pacientes com IAMCSST.^17^ No presente estudo, comparamos os efeitos dessa estratégia em parâmetros bioquímicos e de RMC entre pacientes idosos (até 75 anos de idade) e pacientes não idosos. De fato, no estudo, mediante tratamento e insulto isquêmico similares, não houve diferença na massa infartada entre pacientes idosos e não idosos, sendo que os idosos apresentaram melhor função ventricular. A análise de regressão linear multivariada mostrou que nenhum dos marcadores clínicos e laboratoriais que se diferiram entre idosos e não idosos foram relacionados à FEVE. O tempo de perfusão mais curto foi associado com melhor fluxo coronário, menos remodelamento ventricular adverso e menor mortalidade.^18^ Ainda, em indivíduos com IAMCSST, um blush miocárdico deficiente relacionou-se com mortalidade.^19^ No entanto, esses parâmetros não foram diferentes entre idosos e não idosos, incluindo vasos colaterais.

Os principais determinantes da massa infartada em D1 foram TNTus e PCRus. Ambas as variáveis foram comparáveis em idosos e não idosos e, conforme o esperado, o tamanho do infarto também não foi diferente entre os grupos. Alguns marcadores inflamatórios foram diferentes entre esses grupos, tais como níveis mais altos de IL-18 em D1 e D30 entre os idosos. A interleucina-18 possui similaridades com a IL-1 beta, tais como ativação da caspase-1. No entanto, diferentemente da IL-1 beta, a IL-18 secretada em resposta à ativação de agentes inflamatórios no tecido miocárdico devido à isquemia/perfusão também estimula respostas anti-inflamatórias de defesa.^20 , 21^

Outra diferença entre idosos e não idosos foi a RNL em D30. Em indivíduos submetidos à ICP, a RNL tem sido considerado um marcador de lesão no miocárdio e de disfunção miocárdica. Ainda, uma meta-análise mostrou que a RNL relacionou-se com mortalidade.^22 , 23^ Não houve diferença entre idosos e não idosos quanto à RNL em D1, e esse marcador não foi preditor da massa infartada, possivelmente devido à realização da reperfusão em tempo hábil e à exclusão de indivíduos com idade superior a 75 anos. A análise de regressão linear mostrou que a idade, como variável contínua, não foi preditora de massa infartada ou FEVE.

Os principais determinantes da FEVE foram TNTus, PCRus, e glicemia, mas não foi observada diferença para esses parâmetros entre idosos e não idosos em D1. Em, D30, creatinina e HDL colesterol foram preditores de tamanho do infarto, mas somente a creatinina manteve relação com FEVE. A creatinina sérica foi um pouco mais alta nos idosos, ao passo que o HDL colesterol não foi diferente em D30.

Evidências recentes sugerem que as células B possam exercer um papel relevante na aterosclerose e durante a recuperação do miocárdio após um infarto agudo do miocárdio.^24 , 25^ Nos idosos, a imunossenescência, envolvendo populações celulares e excesso de citocinas, tem sido chamada de “inflamm-aging” em inglês (inflamação relacionada ao envelhecimento).^26^ A maioria das células B e T foram comparáveis entre idosos e não idosos, provavelmente devido ao limite de idade no estudo. Ainda, não foram encontradas diferenças para a maioria dos marcadores inflamatórios, incluindo a PCRus, entre idosos até 75 anos e não idosos.

Como a melhor FEVE entre os idosos não pôde ser explicada por presença de vasos colaterais, outros mecanismos devem estar envolvidos. Pacientes não idosos geralmente apresentam melhor função endotelial e maior atividade fibrinolítica dependente do endotélio que pacientes idosos, o que poderia levar a um tempo mais longo até a completa oclusão coronária, com disfunção da microcirculação dada à maior quantidade de microembolização trombótica.^27^

### Pontos fortes e limitações

Este estudo abordou pacientes idosos com IAMCSST de até 75 anos de idade, uma população geralmente pouco estudada em ensaios clínicos com estratégia farmacoinvasiva, e mostrou um perfil bioquímico comparável ao de pacientes mais jovens. Diferenças nas repostas inflamatórias entre idosos e não idosos foram pequenas, e não tiveram impacto sobre os parâmetros de RMC analisados. Resultados de segurança devem ser interpretados com cautela, uma vez que o número de pacientes é insuficiente para conclusões. Por fim, os resultados aplicam-se somente para pacientes com IAMCSST com reperfusão em tempo adequado (até seis horas do início dos sintomas) e encaminhados para angiografia coronária ou ICP nas primeiras 24 horas.

## Conclusões

A estratégia farmacoinvasiva precoce em idosos de até 75 anos foi associada com tamanho do infarto comparável e melhor função ventricular esquerda que em pacientes não idosos. Até essa idade, pequenas diferenças em marcadores inflamatórios não afetaram os parâmetros de RMC analisados.

### Destaques

- O desempenho da estratégia farmacoinvasiva em pacientes idosos de até 75 anos de idade
- Pacientes idosos e não idosos apresentaram tamanho de infarto similar mediante estratégia fármaco-invasiva similar
- Pequenas diferenças nos marcadores vasculares e inflamatórios entre idosos e não idosos não tiveram relação com a massa infartada
- Pacientes idosos apresentaram melhor fração de ejeção ventricular esquerda que pacientes não idosos
