# Supplementary material for: Estratégia Farmacoinvasiva em Idosos até 75 Anos ou Não Idosos: Análise de Parâmetros Bioquímicos e de Ressonância Nuclear Magnética Cardíaca
Source: Arq Bras Cardiol. 2022 Dec 20;120(1):e20220177. [Article in Portuguese] doi: 10.36660/abc.20220177 (PMC9833293; doi:10.36660/abc.20220177)
Supplement: Translation

# Translation: Pharmacoinvasive Strategy in Elderly Up to 75 Years or Non-Elderly: Analysis of Biochemical and Cardiac Magnetic Resonance Imaging Parameters

**Authors:** Amanda S. Bacchin, Francisco A. H. Fonseca, Rui Povoa, Gilberto Szarf, Ibraim Masciarelli Pinto, Adriano Mendes Caixeta, Daniela Teixeira, Ieda Longo Maugeri, Mayari E. Ishimura, Maria E. R. Coste, Henrique Tria Bianco, Carolina N. França, Maria Cristina Izar

**Affiliations:** 1 Brazil Universidade Federal de São Paulo, São Paulo, SP – Brazil; 2 Brazil Escola Paulista de Medicina da Universidade Federal de São Paulo, Santana de Parnaíba, SP – Brazil; 3 Brazil Instituto Dante Pazzanese de Cardiologia – Cardiovascular CT/MR, São Paulo, SP – Brazil; 4 Brazil Universidade de Santo Amaro – Pós-Graduação, São Paulo, SP – Brazil

**Keywords:** ST Elevation Myocardial Infarction, Cytokines, Lymphocytes

## Abstract

**Background:**

Pharmacoinvasive strategy is an alternative when primary percutaneous coronary intervention (PCI) is not feasible.

**Objectives:**

This study aimed to evaluate the effects of early pharmacoinvasive strategy on the infarct size and left ventricular ejection fraction in elderly and non-elderly patients. The role of inflammatory markers was also examined.

**Methods:**

Patients (n=223) with ST segment elevation myocardial infarction (STEMI) were prospectively included and submitted to pharmacological thrombolysis in the first six hours, and underwent coronary angiogram and PCI when necessary, in the first 24 hours. Blood samples were collected in the first day (D1) and after 30 days (D30). Cardiac magnetic resonance imaging (cMRI) was performed at D30. Significance was set at p<0.05.

**Results:**

Elderly and non-elderly patients showed similar percentage of infarcted mass (13.7 [6.9-17.0] vs. 14.0 [7.3-26.0], respectively, p=0.13) (median [interquartile range]). However, elderly patients had better left ventricular ejection fraction (53 [45-62] vs. 49 [39-58], p=0.025). Titers of interleukin (IL)1beta, IL-4, IL-6, and IL-10 did not differ between D1 and D30, but elderly patients had higher titers for IL-18 at D1 and D30. Absolute numbers of B and T lymphocytes were similar in both groups at D1 and D30, but elderly patients had higher neutrophil/lymphocyte ratio at D30. Multivariate linear regression analysis of cMRI outcomes in the whole population showed that the independent predictors were not different between elderly and non-elderly patients.

**Conclusion:**

Pharmacoinvasive strategy in elderly patients was associated with small differences in inflammatory parameters, similar infarct size and better left ventricular function than non-elderly patients.

## Introduction

Mortality from ST segment elevation myocardial infarction (STEMI) is higher in elderly patients than non-elderly.^1 , 2^Delays in diagnosis and thrombolytic or percutaneous therapies determine greater loss of myocardium and ventricular remodeling, with prognostic implications.^3 , 4^Higher prevalence of cardiovascular risk factors, such as diabetes and hypertension, and severe coronary heart disease increase the challenge in the treatment of elderly patients.^5 - 7^

Pharmacoinvasive strategies have emerged as a great opportunity to reduce the time for coronary reperfusion, allowing a suitable window for complementary percutaneous or surgical treatment.^8 , 9^In addition, more efficient coronary blood flow supply by collateral vessels may increase the recovery of the ischemic myocardium.^10^However, due to immunosenescence, greater inflammatory responses in elderly patients may play additional role during reperfusion and myocardial recovery.^11 , 12^Finally, in the long term after STEMI, recurrence of coronary events and cardiovascular deaths appear to be related to the amount of myocardial necrosis and degree of ventricular impairment.^13 , 14^

Our study aimed to compare the efficacy of pharmacoinvasive strategy in the amount of the infarcted mass and ventricular function, evaluated by cardiac magnetic resonance imaging (cMRI) in elderly (up to 75 years old) and non-elderly patients with STEMI. The study also evaluated the role of inflammatory markers on these cMRI parameters.

## Methods

### Study population

Consecutive patients of both sexes with their first myocardial infarction were included. All patients were submitted to pharmacological thrombolysis with tenecteplase in the first six hours of symptom onset followed by coronary angiography and percutaneous coronary intervention (PCI) when needed, in the first 24 hours of STEMI. These patients received standard-of-care therapies (including highly effective lipid-lowering drugs and dual antiplatelet therapy). Patients with clinical instability, autoimmune disease, known malignancy, signs of active infections, or older than 75 years, were excluded. The exclusion of patients older than 75 years was due to drug-therapy-related safety issues. The study protocol followed the Declaration of Helsinki and was approved by the local ethics committee (CAAE: 38692514.1.1001.5505; IRB 1.253.088). Written informed consent was provided by all subjects before their inclusion.

### Laboratory assays

All patients were referred to our hospital after thrombolysis for coronary angiography during the first 24 hrs of myocardial infarction. Blood samples were collected on the first day of hospitalization, except for patients transferred overnight. In this case, samples were collected in the following morning. Samples were collected on the first day (D1) and after 30 days of STEMI (D30).

Routine laboratory assays were performed at the central laboratory of the university hospital. High-sensitivity C-reactive protein (hsCRP) was measured by immunonephelometry, and B and T subtypes of lymphocytes were determined as previously reported.^12^Briefly, the cells were thawed and diluted in RPMI medium. Cells were stained with fluorescent-conjugated monoclonal antibodies for evaluation of B and T lymphocytes. B1 lymphocyte population was defined as CD3^-^CD19^+^CD20^+^CD27^+^CD43^+^, and B2 lymphocyte subtypes as CD3^-^CD19^+^CD20^+^CD43^-^. T lymphocytes were defined as CD4^+^(CD3^+^CD4^+^CD8^-^) or CD8^+^(CD3^+^CD4^-^CD8^+^).

Circulating interleukins were determined by enzyme-linked immunosorbent assay (ELISA) using specific kits for IL-4, IL-6 and IL-10 (BD Pharmingen-USA) and R&D Systems kits (Minneapolis, USA) for IL-1β and IL-18.^15^

### Cardiac magnetic resonance imaging

Cardiac MRI was performed on the 30^th^day after STEMI to quantify the infarcted mass and to explore the effects of inflammatory variables during recovery and healing of myocardial infarction.^16^Images were performed on 3.0 T scanners and protocol included steady-state free precession (SSFP) imaging for anatomical evaluation, retrospective cine imaging in long and short axis views and late contrast-enhanced imaging using gadolinium for evaluation of myocardium scar/fibrosis as previously reported.^12^The mass of necrotic tissue was estimated in grams and as the percentage of the necrotic tissue of the left ventricular mass.

### Statistical analysis

Categorical variables were reported as number and percentage (%) and compared by Pearson’s chi-square test. Continuous variables were examined for normality using the Kolmogorov-Smirnov test and expressed as median (interquartile range [IQR]) due to their non-Gaussian distribution. The non-parametric Wilcoxon rank sum test was used to compare continuous variables at D1 and D30, and the Mann-Whitney U test to compare continuous variables between elderly and non-elderly patients. Uni- and multivariable linear regression analyses were performed to identify independent predictors for the infarcted mass and left ventricular ejection fraction (LVEF). All the assumptions for the use of linear regression analysis were examined. A convenience sample was used in the study. Statistical analysis was performed using SPSS Statistics 17.0 and significance was set at p < 0.05.

## Results

A total of 322 patients with STEMI were screened and 270 enrolled in the study from May 2015 to March 2019. Forty-seven patients were excluded for the cMRI analysis in the first month, due to mortality, hospitalization or renal insufficiency ( Figure 1 ). During the first month, the rate of cardiovascular events composed of re-hospitalizations due to heart failure, unstable angina, resuscitated sudden death, urgent PCI, and total mortality, did not differ between groups (p=0.49, Chi-square test). All these cardiovascular events had similar distribution between elderly and non-elderly, except for mortality – four elderly patients and three patients in the non-elderly group (p=0.017, chi-square test). Other cardiovascular events such as elective PCI, elective surgical revascularization and bleeding did not differ between these groups.

**Figure 1:** Study flowchart. Stable patients with first myocardial infarction were included. Fifty-two patients were not eligible and 47 were excluded due to death, hospitalizations or contra-indication for cardiac magnetic resonance imaging (cMRI). A total of 223 elderly and non-elderly patients completed the study and performed cMRI after 30 days of acute myocardial infarction. UA: unstable angina; CABG: coronary artery bypass grafting; HF: heart failure; cMRI: cardiac magnetic resonance imaging.

Table 1 shows the main characteristics of the studied population (n=223). Location of myocardial infarction, high-sensitivity troponin T levels (hsTNT), coronary blood flow (TIMI flow grade), collateral vessels (Rentrop grades) and microcirculation (myocardial blush grade) were similar in both groups.

**Table 1:** Main characteristics of the study population

Parameters	Non-elderly (<65 years) N=180	Elderly (65-75 years) N=43	p value
Age, years	54 (48-59)	67 (66-70)	<0.001
Male gender	131 (73)	27 (63)	0.18
Diabetes	47 (26)	10 (23)	0.82
Hypertension	92 (51)	17 (40)	0.85
Smoking	86 (48)	14 (33)	0.004
Body mass index, Kg/m^2^	26.6 (25.6-29.7)	25.5 (23.1-28.6)	0.13
SBP, mm Hg	124 (110-137)	130 (121-140)	0.10
DBP, mm Hg	77 (70-90)	78 (70-86)	0.98
GFR, ml/min/1.73m^2^	93.5 (81.0-100.0)	77.0 (65.0-88.0)	<0.001
hsTNT, U/L	5037 (2238-10089)	4298 (1161-11673)	0.36
**Type of MI**			**0.41**
Anterior	88 (52)	17 (40)	
Inferior	70 (41)	22 (51)	
Lateral	11 (7)	4 (9)	

*Values are median (interquartile range) or frequencies (%); SBP: systolic blood pressure; DBP: diastolic blood pressure; GFR: glomerular filtration rate (estimated by CKD-EPI); hsTNT: high-sensitivity troponin T; MI: myocardial infarction. Continuous variables were compared by the Mann-Whitney U test; categorical variables were compared by Pearson’s chi-square test.*

### Laboratory parameters

Table 2 shows that elderly and non-elderly individuals had comparable laboratory parameters, except for higher HDL-C, neutrophil-to-lymphocyte ratio (NLR) and serum creatinine in the elderly group.

**Table 2:** Laboratory parameters at baseline (D1) and after 30 days (D30), by group

Parameters	Non-elderly (< 65 years) N=180	Elderly (65-75 years) N=43	p value
**D1**			
WBC	12000 (9640-13675)	11000 (9160-12300)	0.07
Lymphocytes	2098 (1532-2725)	1647 (1298-2558)	0.11
NLR	4.63 (3.10-6.78)	4.74 (3.20-7.47)	0.80
Glucose	121 (99-151)	119 (107-142)	0.75
HbA1c	5.9 (5.6-6.6)	6.0 (5.5-6.5)	0.73
Total cholesterol	198 (173-230)	198 (166-232)	0.75
LDL-C	129 (107-154)	123 (103-154)	0.57
HDL-C	41 (33-46)	44 (39-55)	0.03
Triglycerides	133 (97-203)	113 (79-172)	0.10
Non HDL-C	159 (136-193)	151 (120-175)	0.30
Lp (a)	16 (9-41)	19 (13-46)	0.20
Creatinine	0.86 (0.74-1.01)	0.91 (0.78-1.10)	0.06
GFR	94 (81-100)	77 (65-88)	<0.001
hsCRP	21.9 (9.5-48.1)	18.1 (10.8-33.2)	0.63
**D30**			
WBC	7740 (6500-9050)	7710 (6630-8830)	0.94
Lymphocytes	2044 (1590-2441)	1823 (1468-2210)	0.06
NLR	2.77 (2.25-3.48)	3.50 (2.52-4.23)	0.04
Glucose	99 (90-112)	99 (92-115)	0.91
Total cholesterol	123 (106-146)	121 (105-156)	0.85
LDL-C	61 (46-83)	61 (47-84)	0.93
HDL-C	37 (31-44)	39 (34-47)	0.23
Triglycerides	127 (97-164)	128 (104-163)	0.97
Non HDL-C	84 (67-109)	84 (69-113)	0.74
Lp (a)	14 (8-37)	16 (11-38)	0.20
Creatinine	0.93 (0.82-1.08)	1.05 (0.86-1.22)	0.02
GFR	85 (73-87)	68 (59-79)	<0,001
hsCRP	1.99 (0.88-5.27)	3.03 (1.35-7.29)	0.08

*Values are median (IQR). WBC: white blood cells (cells/mm3); NLR: neutrophil to lymphocyte ratio; HbA1c: glycated hemoglobin (%); hsCRP: high-sensitivity C-reactive protein (mg/L); GFR: glomerular filtration rate (CKD-EPI, mL/min/1.73m2). Lp (a): lipoprotein (a). Lipids, glucose and creatinine are mg/dL. Comparisons were made by the Mann-Whitney U test.*

At D1 and D30, the number of T lymphocytes CD4, CD8, as well as B lymphocytes, B1 or B2 subtypes, were comparable in the elderly and non-elderly groups. In addition, the absolute number of classic B2 (naïve plus memory) lymphocytes did not differ between groups ( Table 3 ).

**Table 3:** B and T lymphocytes subtypes at baseline (D1) and after 30 days (D30)

Parameters	Non-elderly (< 65 years) (n=164)	Elderly (65-75 years) (35)	p value
**D1**			
T CD4	871 (567-1198)	1076 (597-1625)	0.40
T CD8	345 (226-484)	307 (188-799)	0.99
B1	5.0 (3.0-11.0)	5.5 (3.3-13.0)	0.58
B2 naïve	53.4 (16.0-115.9)	52.2 (9.89-115.4)	0.58
B2 memory	49.9 (23.4-129.0)	59.5 (27.1-113.3)	0.72
B2 classic	135.1 (75.6-248.3)	124.6 (81.5-226.8)	0.84
**D30**			
T CD4	912 (620-1093)	976 (730-1204)	0.23
T CD8	313 (197-479)	310 (197-552)	0.80
B1	4.2 (2.4-8.1)	2.6 (2.0-5.0)	0.23
Naïve B2	38.5 (11.6-75.0)	59.9 (15.5-104.5)	0.22
Memory B2	38.6 (20.1-85.6)	57.7 (35.9-94.6)	0.07
Classic B2	97.4 (53.6-159.6)	122.0 (87.4-195.6)	0.08

*Lymphocytes as cells/mL. Classic B2 = naïve B2 plus memory B2. Comparisons were made by the Mann-Whitney U test.*

Compared to D1, higher titers at D30 were found for IL-4 (p=0.007) and IL-10 (p<0.001), lower titers for IL-18 and no significant changes for IL-1beta (p=0.058) or IL-6 (p=0.77). Differences between elderly and non-elderly patients at D1 and D30 were observed only for IL-18 ( Figure 2 ).

**Figure 2:** Boxplots of circulating interleukins (IL) at baseline (D1) and 30 days after acute myocardial infarction (D30). (A) Similar concentrations of IL-1β, IL-4, IL-6, IL-10 were observed for elderly and non-elderly patients at D1; (B) At D30, comparable titers of IL-1β, IL-4, IL-6, IL-10 were observed; (C) IL-18 levels were higher among elderly at D1 (p=0.017 vs. non-elderly), and D30 (p=0.023 vs. non-elderly). All analyses were made by the Mann-Whitney U test; *significant differences.

### Cardiac magnetic resonance imaging (cMRI)

Figure 3 shows the parameters of cMRI for elderly and non-elderly patients. Infarct size was comparable between groups and better LVEF was seen among the elderly.

**Figure 3:** Boxplots of cardiac magnetic resonance imaging parameters in elderly and non-elderly patients at D30. (A) Similar infarct size was observed for elderly and non-elderly patients (p=0.25); (B) Better left ventricular ejection fraction (LVEF) was observed for elderly (p=0.02); Mann-Whitney U test; *significant diferences.

Left ventricular (LV) mass (grams) (105 [82-122] vs. 103 [85-123] p=0.78 for elderly and non-elderly patients, respectively), as well as the percentage of the LV fibrosis (13.7 [6.9-17.0] vs. 14.0 [7.3-26.0], p=0.13 for the elderly and non-elderly patients, respectively) were not different between the groups.

Both LVEF (%) and right ventricular ejection fraction (59 [52.0-67.0] vs. 55.0 [50.0-62.0], p=0.012) were higher in the elderly compared with non-elderly patients at D30. The percentage of patients with LVEF ≤ 40% was similar in both groups of patients (chi-square 1.39, p=0.24).

### Multivariable linear regression

Univariable analysis revealed that hsTNT, hsCRP, systolic blood pressure, NLR, creatinine, and weight were significantly associated with the infarct size (grams) at D1 ( Table 4 ). Multivariable linear regression analysis (ANOVA p<0.001) identified hsTNT (beta coefficient 0.392, p<0.001) and hsCRP (beta coefficient 0.299, p<0.001) as independent predictors. For LVEF, univariable regression analysis at D1 showed a significant association with hsTNT, hsCRP, NLR, white blood cells (WBC), glycemia, and lymphocytes ( Table 4 ). Multivariable linear regression analysis at D1 (ANOVA p<0.001) identified hsTNT (beta coefficient -0.367, p<0.001), hsCRP (beta coefficient -0.273, p<0.001), and glycemia (beta coefficient -0.162, p=0.018) as independent predictors.

**Table 4:** Univariable linear regression analysis

	Infarcted mass (grams)		LVEF (%)	

	r	p value	r	p value
**At D1**				
hsTNT	0.380	<0.001	0.428	<0.001
hsCRP	0.331	<0.001	0.319	<0.001
NLR	0.153	0.038	0.296	<0.001
SBP	0.171	0.022	0.135	0.062
Creatinine	0.152	0.029	0.038	0.575
Weight	0.139	0.046	0.035	0.608
WBC	0.099	0.178	0.231	0.001
Glycemia	0.049	0.586	0.162	0.016
Lymphocytes	0.144	0.051	0.178	0.012
**At D30**				
Creatinine	0.272	<0.001	0.214	0.002
WBC	0.179	0.015	0.066	0.353
B2 naïve	0.207	0.024	0.154	0.080
CD4 T	0.267	0.023	0.134	0.239
CD8 T	0.273	0.020	0.265	0.018
Total cholesterol	0.101	0.153	0.160	0.019
Non-HDL-C	0.048	0.503	0.150	0.032
HDL-C	0.209	0.004	0.160	0.019

*hsTNT: high sensitivity troponin; hsCRP: high sensitivity C-reactive protein; NLR: neutrophil/lymphocyte ratio; WBC: white blood cell; B and T lymphocytes were quantified as cells/mL; r: coefficient of correlation; D1: samples collected at the first day of myocardial infarction; D30: samples collected 30 days after myocardial infarction; LVEF: left ventricular ejection fraction. Infarcted mass and left ventricular ejection fraction were determined by cardiac magnetic resonance imaging at D30.*

At D30, univariable regression analysis showed the following variables associated with infarct size: creatinine, B2 naïve lymphocytes, CD4 T lymphocytes, CD8 T lymphocytes, HDL-C, and WBC ( Table 4 ). Multivariable regression analysis at D30 (ANOVA p<0.001) showed creatinine (beta coefficient 0.247, p=0.001) and HDL-C (beta coefficient -0.172, p=0.017) as independent predictors of infarcted mass. For LVEF, among the variables collected at D30, the following were associated in univariable regression analysis: CD8 T lymphocytes, creatinine, non-HDL-C, total cholesterol ( Table 4 ). After multivariable regression analysis, creatinine remained an independent predictor for LVEF (ANOVA p=0.002, beta coefficient -0.211).

## Discussion

Pharmacoinvasive strategy has been considered an efficient alternative to primary PCI for patients with STEMI.^17^In the present study, we compared the effects of this strategy in biochemical and cMRI parameters between elderly patients up to 75 years old and non-elderly patients. In fact, in the study, under similar ischemic insult and treatment, elderly and non-elderly patients had the same amount of infarcted mass, with elderly patients showing better ventricular function. Multivariable linear regression analysis showed that none of the clinical and laboratory markers that differed between elderly and non-elderly patients were related to LVEF. Shorter reperfusion time has been associated with better coronary blood flow, less adverse ventricular remodeling and lower mortality.^18^Furthermore, in subjects with STEMI, impaired myocardial blush has been related to mortality.^19^However, these parameters did not differ between elderly and non-elderly patients, including coronary collateral vessels.

Main determinants of the infarcted mass at D1 were hsTNT and hsCRP. Both variables were comparable among elderly and non-elderly and, as expected, similar infarct size was observed. Some inflammatory markers differed between these groups, such as, the higher IL-18 levels at D1 and D30, among the elderly. Interleukin-18 shares some similarities with IL-1beta, such as its activation by caspase-1. However, unlike IL-1beta, IL-18 released in response to activation of inflammasome in myocardial tissue due to ischemia/reperfusion, may also stimulate anti-inflammatory responses for host defense.^20 , 21^

Another difference between elderly and non-elderly was NLR at D30. In subjects undergoing PCI, NLR has been considered a marker of myocardial injury and myocardial dysfunction, and a meta-analysis showed that NLR was related to mortality.^22 , 23^No differences were observed between elderly and non-elderly for NLR at D1, and this marker was not predictor of infarcted mass, possibly due to timely reperfusion and exclusion of subjects over the age of 75. Linear regression analysis showed that age, as a continuous variable, did not predict infarcted mass or LVEF.

Main determinants of LVEF were hsTNT, hsCRP, and glycemia, but no differences were noted for these parameters between elderly and non-elderly at D1. At D30, creatinine and HDL-C were predictors of infarct size, but only creatinine remained related to LVEF. Serum creatinine was slightly higher in the elderly, while HDL-C did not differ at D30.

Recent evidence suggests that B cells may play a relevant role in atherosclerosis and during the myocardial healing after acute myocardial infarction.^24 , 25^In the elderly, the immunosenescence, involving cell populations and excess of cytokines release, has been called inflamm-aging.^26^Most B and T cells were comparable between elderly and non-elderly, probably due to the limit of age in the study. Furthermore, no differences were found for most inflammatory markers, including hsCRP, between elderly patients up to 75 years and non-elderly ones.

As collateral vessels did not explain the better LVEF among the elderly, other mechanisms should be involved. Non-elderly patients usually show better endothelial function and greater endothelial-dependent fibrinolytic activity than the elderly, which could lead to longer time until complete coronary occlusion, with impairment of microcirculation due to greater amount of thrombotic microembolization.^27^

### Strengths and limitations

This study addressed elderly STEMI patients up to 75 years old, a population usually not reported in clinical trials on pharmacoinvasive strategies, and showed a biochemical profile comparable to younger patients. Differences in the inflammatory responses between elderly and non-elderly patients were small and did not impact on the cMRI parameters analyzed. Safety results should be interpreted with caution as the number of patients included is insufficient for conclusions. Finally, the results apply only for STEMI patients with timely reperfusion (<6 hours of symptoms onset) and referred for coronary angiography or PCI in the first 24 hours.

## Conclusions

Early pharmacoinvasive strategy in elderly patients up to 75 years was associated with comparable infarct size and better LV function than non-elderly patients. Until this age, small differences in inflammatory markers did not affect the cMRI parameters analyzed.

### Highlights

- The performance of pharmacoinvasive strategy in elderly patients up to 75 years old has been little reported
- Elderly and non-elderly patients had similar infarct size under pharmacoinvasive strategy
- Small differences in vascular and inflammatory markers between elderly and non-elderly patients did not account for the amount of infarcted mass
- Elderly patients showed better left ventricular ejection fraction than non-elderly patients
